# Anticonvulsant Activity of *Argyreia speciosa* in Mice

**DOI:** 10.4103/0250-474X.54277

**Published:** 2009

**Authors:** N. S. Vyawahare, S. L. Bodhankar

**Affiliations:** AISSMS College of Pharmacy, Kennedy Road, Near RTO, Pune-411 001, India; 1 Bharati Vidyapeeth Deemed University's Poona College of Pharmacy, Erandwane, Pune-411 038, India

**Keywords:** Pentylenetetrazole, seizures, clonus, *Argyreia speciosa*, maximal electroshock-induced convulsions

## Abstract

*Argyreia speciosa* commonly known as *Vridha daraka* in Sanskrit is one of the important plants used in indigenous system of medicine. The root is regarded as an alternative tonic and useful in the diseases of nervous system. To confirm the veracity of aforementioned claim, we have evaluated the anticonvulsant effect of the extract. In this investigation, the mice were pretreated with different doses of *Argyreia speciosa* extract (100, 200, 400 mg/kg) for 10 days and then, they were subjected to either pentylenetetrazole (80 mg/kg) or maximal electroshock seizures (50 mA, 0.2 s) treatment. The hydroalcoholic extract of *Argyreia speciosa* at the dose of 200 and 400 mg/kg significantly delayed the latency to the onset of first clonus as well as onset of death in unprotected mice and exhibited protection in 16.66% and 33.33% of pentylenetetrazole treated mice respectively. Whereas in case of maximal electroshock-seizures, the dose of 200 and 400 mg/kg significantly reduced the duration of hind limb extension and both the doses were statistically found to be equipotent. The reference standards, clonazepam (0.1 mg/kg) and phenytoin (20 mg/kg) provided complete protection. Thus, present study revealed anticonvulsant effect of *Argyreia speciosa* against pentylenetetrazole- and maximal electroshock-induced convulsions in mice.

*Argyreia speciosa* (AS), commonly known as elephant creeper, is a native of Bengal but found throughout India except in dry western regions up to 1000 feet elevation. It is cultivated in the gardens as an ornamental plant for its green leaves and beautiful rose purple flowers. It is also extensively used in the indigenous system of medicine[1,2]. In India, the leaves and roots of the plants are usually employed for its medicinal use[1]. The plant is prescribed in gonorrhea, strangury and chronic ulcers. The leaves are used externally in the treatment of ringworm, eczema, itch and other skin diseases and are used internally to cure boils and swellings[3]. The roots are recommended as aphrodisiac, diuretic and also in rheumatism[1]. It is one of the constituents in the polyherbal preparations named Fortege[4] and Geriforte[5,6] recommended for curing sexual disorders in male and as an energizer, respectively.

The alcoholic and aqueous extract of leaves of *Argyreia speciosa* reported for dose dependant pregnancy interruption action in rats at early stages of pregnancy[7]. Another study reported dose dependent potentiation of delayed type of hypersensitivity reaction mediated by enhanced production of circulating antibody titre. This action was attributed to flavonoids[8]. Ethanol extract of *Argyreia speciosa* roots has also been documented for antiinflammatory and antiarthritic activity in carrageenan and Freund's complete adjuvant models, respectively. The study further suggested the possible role of flavonoid and triterpene components[9]. The major constituents isolated are friedelin, ergine, agroclavine, penniclavine, chanclavine, ergometrine, quercetin, kaempferol[10], scopoletin, hexadecanyl *p*-hydroxycinnamate[11]. Roots are also claimed to be useful in the disorders of nervous system[1]. To the best of our knowledge no reports are available on its anticonvulsant activity. Hence it was considered worthwhile to investigate the anticonvulsant effect of *Argyreia speciosa* against seizures induced by pentylenetetrazole and maximal electroconvulsive shock in mice.

Hydroalcoholic extract of AS prepared by the procedure mentioned below was received as gift sample (No: RG/4019) from Green Chem., Bangalore, India. Roots were extracted with 50% aqueous alcohol and concentrated. The concentrated mass was washed with petroleum ether several times to remove the resinous matter and fats. Then, the mass was diluted with 25% aqueous alcohol, filtered and concentrated, dried to get a powder form of the extract.

Different doses (100, 200, 400 mg/kg; p.o.) of this extract were administered to the male Swiss mice (20-22 g) maintained at our animal house under standard environmental conditions and diet. The study protocol was approved by Institutional Animal Ethical Committee. Pentylenetetrazole (Sigma) was procured from a local vendor while phenytoin (Eptoin, Abbott India Ltd) and clonazepam (Clonotril, Torrent Pharma) were purchased from a local pharmacy.

Animals were divided into five groups each consisting of six mice and treated for 10 days. This treatment schedule was selected from our previous pilot studies. On 10^th^ day, 60 min after administration of last dose of AS, clonic seizures and tonic clonic convulsions were induced in mice by subcutaneous injection of pentylenetetrazole (80 mg/kg) and by giving maximal electroshock seizures (50 mA for 0.2 s) using an electroconvulsiometer[12], respectively. The latency to the onset of first clonus in unprotected mice and lethality during the following 24 h in pentylenetetrazole treated mice was recorded[13,14]. While the percentage of animals protected from tonic hind limb extension seizure (abolition of tonic hind limb extension within 10 s after delivery of the electroshock were considered as a protected mice) and duration of tonic hind limb extension in unprotected mice subjected to maximal electroshock seizures[15] was recorded. These parameters were compared with those of control mice using one way ANOVA followed by Dunnett's t test to assess the anticonvulsant activity of the extract.

Pentylenetetrazole produced clonic convulsions as well as lethality in mice, whereas pretreatment of the mice with the different doses of AS extract caused significant delay in the onset of the convulsions as well as lethality. At a dose of 100 mg/kg, AS exhibited no significant effect on the latency to the onset of convulsions or number of deaths. However, it prolonged the onset of lethality from 5.09±0.22 min to 5.68±0.27 min (Table 1). The other two higher doses (200, 400 mg/kg) delayed the latency to the onset to 6.03±0.28 min and 6.99±0.07 min, respectively. Moreover, it also significantly prolonged the onset of the lethality after pentylenetetrazole administration and showed protection in 16.66% and 33.33% mice, respectively. The reference standard clonazepam exhibited complete protection. In case of maximal electroshock seizures; the electroshock produced hind limb extension in vehicle treated control mice (36.16±1.62 min), which was significantly reduced with pretreatment of AS 200 and 400 mg/kg (fig. 1).

**Fig. 1:**
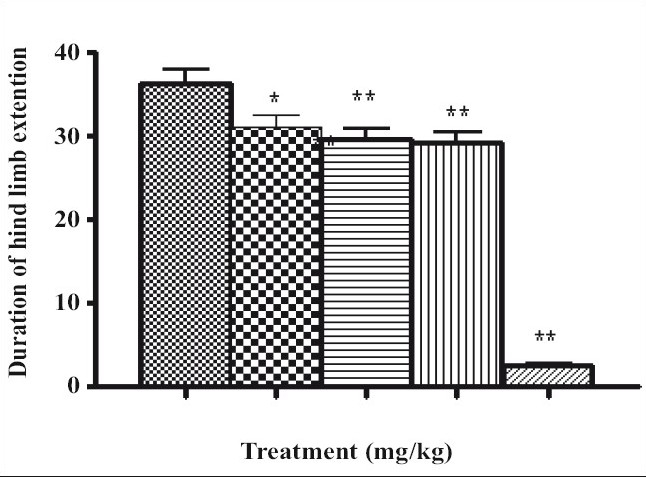
Effect of AS on duration of hind limb extension after MES *Average of six readings. AS is *Argyreia speciosa*, MES is maximal electroshock seizures. Control (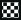), AS-100 (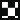), AS-200 (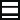), AS-400 (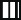) and phenytoin (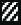)

**TABLE 1:** EFFECT OF AS ON ONSET OF FIRST CLONUS AND TIME OF DEATH AFTER PENTYLENETETRAZOLE

Treatment	Onset of first clonus (m)	Time of death (m)	Incidence of convulsion
Vehicle + PTZ	5.09±0.22	9.72±0.44	6/6
AS 100 + PTZ	5.68±0.27	12.31±0.48*	6/6
AS 200 + PTZ	6.03±0.28*	13.57±0.61**	5/6
AS 400 + PTZ	6.99±0.07**	19.56±1.15**	4/6
Clonazepam + PTZ	-	-	0/6

* Average of six readings. AS is *Argyreia speciosa*, PTZ is pentylenetetrazole

The present study revealed that AS extract possesses significant anticonvulsant activity against pentylenetetrazole and maximal electroshock-induced convulsions. These models are of predictive relevance regarding the clinical spectrum of activity of experimental compounds[13]. Maximal electroshock seizures and pentylenetetrazole tests are assumed to identify anticonvulsant drugs effective against generalized tonic clonic partial seizures and generalized clonic seizures respectively[13,16]. The effect of AS in these tests could therefore suggest anticonvulsant efficacy against the above mentioned types in man. It has often been stated that antiepileptic drug that either prevents or delays pentylenetetrazole induced convulsions, act by elevating the seizure threshold, whereas drugs that block maximal electroshock-induced tonic extension act by blocking spread of seizure[13,17]. Moreover this tonic extension can be prevented either by drug that inhibits voltage-dependent Na^+^ channels, such as phenytoin, valproate, lamotrigine or by drugs that block glutamatergic excitation mediated by the NMDA receptor such as felbamate[18,19]. It is also well established that facilitation of release of glutamate from the nerve terminals and astrocytes results in to glutamate induced neurotoxicity[20]. The scientific validation of its traditional claim as tonic to dullness of intellect[21] and incorporation of AS in the restorative tonic promoted for cognitive enhancement[6] further support its action against glutamatergic transmission. On the other hand, drug that reduces T- type Ca^++^ currents like ethosuximide or drug that enhances GABA_A_ receptor-mediated inhibitory neurotransmission, such as benzodiazepines and phenobarbital elevate the seizure threshold and thereby prevent seizures induced by pentylenetetrazole[22]. Cognitive deficit is one of the major problems associated with epilepsy; underlying pathology as well as drug therapy, and the can lead to disturbances in cognitive function[23]. The present results regarding its anticonvulsant activity have become an added advantage to it being a nootropic[21], and can effectively prevent cognitive deficit associated with convulsions[23].

In the present investigation, AS extract showed significant prevention of pentylenetetrazole as well as maximal electroshock-induced seizures and thereby suggest multiplicity of putative mechanisms of action which might be due to the presence of different phytochemicals in the AS extract interacting simultaneously. The exact mechanisms and their contributions to the anticonvulsant property of AS will be better understood after detailed phytochemical and biochemical analysis, which is underway.

In conclusion, the hydroalcoholic extract of roots of AS possesses anticonvulsant activity against pentylenetetrazole and maximal electroshock seizures induced convulsions and hence may be employed to prevent convulsions and as an adjuvant therapy against cognitive deficit in convulsions.
